# Impact of Safety Nets on Household Coping Mechanisms for COVID-19 Pandemic in Malawi

**DOI:** 10.3389/fpubh.2021.806738

**Published:** 2022-02-07

**Authors:** Martha Mnyanga, Gowokani Chijere Chirwa, Spy Munthali

**Affiliations:** Economics Department, University of Malawi, Zomba, Malawi

**Keywords:** health economics, COVID-19 Malawi, health policy Malawi, public policy Malawi, safety nets, COVID-19 Africa, social protection programs Malawi, COVID-19 urban cash intervention Malawi

## Abstract

**Background:**

Covid-19 pandemic induced various shocks to households in Malawi, many of which were failing to cope. Household coping mechanisms to shocks have an implication on household poverty status and that of a nation as a whole. In order to assist households to respond to the pandemic-induced shocks positively, the government of Malawi, with support from non-governmental organizations introduced Covid-19 Urban Cash Intervention (CUCI) and other safety nets to complement the existing social protection programs in cushioning the impact of the shocks during the pandemic. With these programmes in place, there is a need for evidence regarding how the safety nets are affecting coping. Therefore, this paper investigated the impact that safety nets during Covid-19 pandemic had on the following household coping mechanisms: engaging in additional income-generating activities, receiving assistance from friends and family; reducing food consumption; relying on savings; and failure to cope.

**Methods:**

The study used a nationally representative panel data from the Malawi High Frequency Phone Survey on Covid-19 (HFPS Covid-19) and complemented it with the fifth Integrated Household Panel Survey (IHPS), also known as living standards measurement survey. Five Random Effects Probit Models were estimated, one for each coping mechanism.

**Results:**

Findings from this study indicated that beneficiaries of safety net programs were more likely to rely on remittances from friends and family than the people who had no safety nets. Furthermore, the safety net recipients were less likely to reduce food consumption or rely on savings than the non-recipients. Despite the interesting findings, we also noticed that safety nets had no significant impact on household engagement in other income-generating activities in response to shocks.

**Conclusion:**

The results imply that safety nets in Malawi during the Covid-19 pandemic had a positive impact on consumption and prevented the dissolving of savings. Therefore, these programs have to be scaled up, and the volumes be revised upwards.

## Introduction

Countries, including Malawi, responded in various ways to the novel Covid-19 pandemic that disrupted many economies around the world. Malawi introduced various containment measures to reduce the spread of the virus. These measures, included compulsory screening of all travelers coming into the country at the port of entry, a ban on all travelers from highly affected countries, restrictions on public gatherings to a maximum of 100 people and closure of all schools (1). These measures, coupled with people's fears of the novel virus, partly contributed to the disruptions in the supply chains of various goods and services in the country (1). Business operations were also affected as opening hours were restricted.

As a result, Covid-19 Pandemic induced other shocks to households, communities and nations apart from being a health shock in itself (2–4). According to the National Statistical Office (NSO) of Malawi, the uppermost shocks that the households reported to have experienced from mid-March to July 2020 as a result of Covid-19 pandemic included a fall in the price of farming/business output reported by 66% of the interviewed households. An increase in price of farming/business inputs and disruption of farming, livestock, and/or fishing activities were experienced by 30 and 29% of the households, respectively (5). Whereas, from August 2020 to January 2021, the statistics show that 59% of the interviewed households were affected by an increase in price of major food items consumed, 36% by an increase in price of farming/business inputs and 20% by non-farm business closure (6). This shows that most of the Covid-19 induced household shocks were not idiosyncratic in nature as they likely affected a large proportion of individuals within the sample and beyond.

As such, the affected households no longer turned to each other for assistance or for credit and most of them were finding it hard to cope with the shocks that they faced. For instance, 78% of the households who were hit by one or more shocks from March to July 2020 did nothing in response to these shocks (5). This has negative implications since such households were likely to move into poverty if shocks persisted or they would not be able to move out of it if they were already poor. On top of that, 20 and 31% relied on savings from March to July 2020 and August 2020 to January 2021, respectively (5, 6). Channeling savings into household consumption has long term negative implications on household investment and, therefore that of the nation.

On top of that, reliance on savings is not sustainable, and there was a high probability that such households would move into poverty and fail to cope if shocks persisted. In turn, all these pose a challenge toward eradicating poverty as highlighted in the development agendas such as Sustainable Development Goals (SDGS), Malawi Vision 2063, Pan African Vision 2063, and the Malawi Growth and Development Strategy (7–10). Therefore, it is important to understand how households had been responding to these shocks given that coping responses have an implication on the households' socio-economic well-being and that of a nation as a whole.

Literature shows that health shocks trigger borrowing or assistance from friends and family rather than the use of any other strategy as these shocks are idiosyncratic (11–14). However, evidence of a reduction in these informal-risk sharing coping mechanisms during Covid-19 pandemic has also been reported elsewhere (15). Additionally, others (16) also found evidence that there was less reliance on savings during a lockdown as a result of Covid-19 pandemic. However, these studies did not consider the economic interventions that had been put in place given their recency and the role they played in assisting households cope positively with various shocks.

In order to assist households to respond to the economic crisis due to pandemic positively, the government of Malawi, with support from Non-Governmental Organizations introduced Covid-19 Urban Cash Intervention (CUCI) to complement the existing social protection programs. Safety nets play a big role in replacing lost incomes for households affected by various economic shocks and those facing credit constraints to avoid the use of negative coping mechanisms and improve their consumption and increase their asset holdings (17–20). In this regard, this study was implemented to investigate the impact that safety nets during Covid-19 pandemic had on household coping mechanisms for the pandemic in Malawi.

The study matters and it is of significance to undertake in Malawi at this point and offers lessons beyond Malawi. Firstly, it feeds into the development agenda for Malawi as it potentially shows the aspects that may help put the country on track with the agenda. The Malawi development agenda is guided by the United Nations Sustainable Development Goals (SDGs), whose goal number 1 is to “end poverty in all its forms everywhere.” Under this goal, target 3 stresses the “implementation of nationally appropriate social protection systems and measures for all, including floors, and by 2030 achieve substantial coverage of the poor and the vulnerable.” Target 5 states, “by 2030, build the resilience of the poor and those in vulnerable situations and reduce their exposure and vulnerability to climate related extreme events and other economic, social and environmental shocks and disasters” (7). Therefore, this study established the role of safety nets during Covid-19 pandemic in reducing household vulnerability to shocks and building their resilience to future shocks for the realization of these targets in Malawi. Hence, the study speaks directly to SDGs 1 and 5.

Furthermore, Goal number 2 under the SDGs is to “end hunger, achieve food security and improve nutrition and promote sustainable agriculture.” Target number 1 under this goal states: “by 2030, end hunger and ensure access by all people, in particular, the poor and the people in vulnerable situations, including infants, to safe, nutritious and sufficient food all year round.” Target 2 states: “by 2030, end all forms of malnutrition, including achieving, by 2025, the internationally agreed targets on stunting and wasting in children under 5 years of age, and address the nutritional needs of adolescent girls, pregnant and lactating women, and older persons” (7). Guided by this, the goal on the key priority area of Health and Population Management of the Malawi Growth and Development Strategy (MGDS III), is to “Improve health and quality of the population for sustainable socio-economic development” and one of the outcomes is “reduced morbidity and mortality due to malnutrition” through “promoting dietary diversity and consumption of high nutrient value by addressing the production and marketing bottlenecks particularly of fruits” (9). Therefore, this study established the role of safety nets in offsetting household reduction in food consumption as a response to a shock, since this mechanism has a negative implication on household nutrition status. This also impacts the nutrition status for school-going children, which eventually impacts their learning outcomes and, hence, the potential for a vicious circle of ex-ante child poverty.

All in all, the study contributes to the existing literature on the importance of safety nets during a crisis in achieving the goal of poverty eradication in the medium term through building household resilience to shocks. It also assessed the role of safety nets in enabling household investments to achieve the long-term goal of transforming the Malawi nation into an upper middle-income nation by 2063 as per the aspiration of Malawi vision 2063. This was achieved through an empirical assessment of the impact that safety nets have on the following household coping mechanisms in response to a shock during the pandemic: engaging in additional income-generating activities, receiving assistance from friends and family; reducing food consumption; relying on savings; and failure to cope. In addition, it is the first study within the region and Malawi in particular, which assess the coping aspect in the times of COVID-19.

## Methods

### Data

The paper used survey data of Malawi High Frequency Phone Survey on Covid-19 (HFPS-Covid-19) that was conducted by NSO with support from the World Bank. The survey aimed to monitor the socio-economic impacts of Covid-19 pandemic on households in Malawi. This paper used the second and the third waves of the survey. The study also drew some variables from the fifth Integrated Household Panel Survey. The data is readily available for public use and free to download from the World Bank website. https://microdata.worldbank.org/index.php/catalog/3766.

### Variables

In the second and third waves, the households were asked if they experienced any shock(s) due to Covid-19. Each household would list the shock(s) and one coping mechanism they used in response to each. The study mainly focused on five coping mechanisms that were mostly reported by the households. Therefore, the dependent variables were these coping mechanisms: engaging in additional income generating activities, receiving assistance from friends and family, reducing food consumption, relying on savings, as well as doing nothing. These variables took a value of 1 if the household adopted that particular mechanism and a 0 otherwise.

As explanatory variables, the models included the variable on whether the household was a beneficiary of any social safety net program. The variable was binary, bearing the value of 1 for a beneficiary and a 0 for a non-beneficiary. The models also included variables on household demographic characteristics of; size, number of dependents, and number of household members above 18 years old. Also included were household head characteristics of: age, sex (which took the value of 0 if the household head was male and the value of 1 if the head was female), their education level, their sector of employment (which took the value of 0 if the household head was employed in a non-agricultural sector and 1 if they were employed in the agricultural sector), and marital status (which took the value of 1 for married household heads and 0 for unmarried household heads). Other household characteristics in the model included the wealth category of the household, its place of residence (which took the value of 0 for households in the rural area and 1 for those in the urban area), as well, as region. On top of that, the shock variables categorized into economic shocks, health shocks and socio-political shocks were also included. Also considered in the study as one of the explanatory variables was the scope of the shocks, i.e., whether the shock was an idiosyncratic or covariate. This variable took the value of 1 if the shock was idiosyncratic and 0 otherwise.

### Data Analysis

Firstly, descriptive statistics and cross-tabulations were computed to understand the characteristics of the sample for this study. These are presented in Tables 1–**3**. Then after the descriptive analysis, the econometric analysis was done. Five Random Effects Probit Models were run, because of the binary nature of the outcome variables, one for each coping mechanism. On top of these, gender and regional Random Effects Models were also run to establish if there are heterogeneities in terms of gender and region. Results for the various econometric analysis done, are shown in **Tables 4**–**8**.

**Table 1:** Descriptive statistics.

**Variable**	**Obs**.	**Mean**	**Std. Dev**.	**Min**	**Max**.
Safety nets beneficiary	3,140	5%	0.213	0	1
Age of head	3,140	42	14.225	16	98
Female head	3,140	21%	0.405	0	1
Household size	3,140	5	2.274	1	19
Number of dependents	3,140	2	1.532	0	12
People Above 18 years old	3,140	2	1.164	1	9
Head has no education	3,140	3%	0.169	0	1
Head has primary education	3,140	51%	0.5	0	1
Head has secondary education	3,140	37%	0.484	0	1
Head has tertiary education	3,140	9%	0.287	0	1
Head in married	3,140	77%	0.423	0	1
Agricultural sector	3,140	25%	0.432	0	1
Wealth quintile 1	3,140	32%	0.467	0	1
Wealth quintile 2	3,140	26%	0.44	0	1
Wealth quintile 3	3,140	20%	0.398	0	1
Wealth quintile 4	3,140	13%	0.337	0	1
Wealth quintile 5	3,140	9%	0.285	0	1
Urban	3,140	37%	0.482	0	1
Northern region	3,140	15%	0.356	0	1
Central region	3,140	42%	0.494	0	1
Southern region	3,140	43%	0.495	0	1
Idiosyncratic shock	3,140	51%	0.5	0	1
Economic shock	3,140	72%	0.448	0	1
Health shock	3,140	9%	0.289	0	1
Socio political shock	3,140	17%	0.378	0	1
Engaging in other activities	3,140	2%	0.135	0	1
Receiving assistance from friends and family	3,140	2%	0.126	0	1
Reducing food consumption	3,140	3%	0.156	0	1
Relying on savings	3,140	9%	0.284	0	1
Doing nothing	3,140	39%	0.487	0	1

## Results

### Descriptive Statistics

As shown in Table 1, about 5% of the households in the sample benefited from at least one of the social protection programs. These included free food, Social Cash Transfers (SCTs), CUCI, other cash transfers, as well as other in-kind transfers (excluding food). The table also indicates that the average age of heads of the households that were included in the sample was 42, with the youngest head being 16 and the oldest being 98 years old.

On top of that, the sample largely comprised male headed households, with about 21% of the households being headed by a female. The table also shows that 77% of the household heads in this sample were married while the rest were unmarried. The unmarried category included those who had never been married, those who divorced/separated, and those who were widowed. In terms of household size, the average number of members per household in this sample was five. The smallest household had one member, and the largest had 19 household members.

On top of that, the average number of dependents per household, as well as adults above 18 years old, was two. In addition, about half of the households were headed by a member with primary education (51%), almost 37% had secondary education, whereas 9 and 3% of household heads had tertiary and no education, respectively. The sample also comprised households whose majority were employed in a non-agricultural sector, with about 25% being employed in the agricultural sector. In terms of wealth categories, a large proportion of the households in the sample (32%) were in the lowest quintile, whereas the top quintile consisted of about 9% of the households in the sample.

Regarding location, the majority of the households in this sample lived in the rural areas, with about 37% living in the urban areas. Furthermore, 15% of the households was in the Northern region, 42% was in the central region and 43% was from the southern region. This means the study was dealing with households mainly from the central and southern regions.

In terms of shock distribution, 72% of shocks experienced by the households were economic related shocks which included: job loss, non-farm business closure, increase in price of farming/business inputs, fall in the price of farming/business output, as well increase in the price of major food items consumed. Almost 9% of the household shocks were health shocks which included illness, injury, or death of income-earning member of the household. At the same time, 17% were socio-political shocks which were disruption of farming, livestock and fishing activities as well as theft/looting of cash and other property. On top of that, 51% of all these shocks were idiosyncratic in nature as they only affected one household at a particular time.

Out of the households that experienced at least a shock in this sample, about 39% failed to cope with the shocks, 9% relied on their savings, 3% reduced their food consumption, whereas 2% engaged themselves in other income generating activities, and another 2% received assistance from friends and family. This means the rest used other mechanisms not included in this analysis. This implies that a good number of households were indeed failing to cope with various shocks during this Covid-19 pandemic either because of the magnitude of the shocks or they did not have the capacity to respond to the shocks using any other mechanism.

### Distribution of Coping Mechanisms

Table 2 indicates that the majority of households who indicated to have been engaged in other income generating activities, received assistance from friends and family, reduced food consumption, relied on savings or failed to cope after experiencing a shock were headed by a male member.

**Table 2:** Distribution of coping mechanisms.

	**Engaging**		**Receiving**		**Reducing**		**Relying**		**Doing**	
	**in other activities**		**assistance**		**consumption**		**on savings**		**nothing**	
	**No**.	**%**	**No**.	**%**	**No**.	**%**	**No**.	**%**	**No**.	**%**
**Sex of head**										
Male	47	81	27	53	63	81	229	83	937	77
Female	11	19	24	47	15	19	48	17	272	23
Total	58	100	51	100	78	100	277	100	1,209	100
**Education**										
No education	0	0	1	2	3	4	4	1	49	4
Primary	37	64	27	53	33	42	124	45	673	56
Secondary	16	28	22	43	35	45	111	40	414	34
Tertiary	5	8	1	2	7	9	38	14	73	6
Total	58	100	51	100	78	100	277	100	1,209	100
**Marital status**										
Unmarried	11	19	23	45	14	18	57	21	291	24
Married	47	81	28	55	64	82	220	79	918	76
Total	58	100	51	100	78	100	277	100	1,209	100
**Employment sector**										
Non-agriculture	43	74	43	84	69	89	240	87	815	67
Agriculture	15	26	8	16	9	11	37	13	394	33
Total	58	100	51	100	78	100	277	100	1,209	100
**Region**										
North	20	35	18	35	12	15	25	9	149	12
Central	20	35	16	32	9	12	129	47	519	43
Southern	18	30	17	33	57	73	123	44	541	45
Total	58	100	51	100	78	100	277	100	1,209	100
**Residence**										
Rural	35	60	27	53	46	59	157	57	814	67
Urban	23	40	24	47	32	41	120	43	395	33
Total	58	100	51	100	78	100	277	100	1,209	100
**Wealth**										
Wealth quintile 1	10	17	13	26	38	49	105	38	334	28
Wealth quintile 2	18	31	15	29	17	22	79	29	302	25
Wealth quintile 3	15	26	10	20	5	6	47	17	266	22
Wealth quintile 4	11	19	10	20	8	10	27	9	180	15
Wealth quintile 5	4	7	3	5	10	13	19	7	127	10
Total	58	100	51	100	78	100	277	100	1,209	100

The majority of households headed by someone with primary education were using all the mechanisms except reducing food consumption in response to a shock. On top of that, the majority of those relying on savings were employed in the non-agricultural sector.

In terms of location, those who relied on savings and those who failed to cope with the shocks were mainly from the central and southern region. In contrast, most of those who engaged in additional income-generating activities and those who received assistance were from the northern region. On top of that, more than half of those who reduced their food consumption were from the country's southern region. In addition to that, all the five mechanisms used in the analysis were mainly used by households that lived in the rural areas.

Those who reduced food consumption relied on savings, and failed to cope in response to a shock were mainly the poorest (lowest quintile). At the same time, the majority of those who engaged in other income generating activities and those who received assistance from friends and family were in the second lowest quintile of wealth.

### Distribution of Shocks

In terms of distribution of the three types of shocks, households in the central, southern, and rural areas had been hard hit compared to those in the north and in the urban areas. This is shown in Table 3.

**Table 3:** Distribution of shocks.

	**Economic shocks**		**Health shocks**		**Socio-political shocks**	
	**No**.	**%**	**No**.	**%**	**No**.	**%**
**Region**						
North	300	13	54	19	94	17
Central	958	42	141	49	255	47
Southern	1,008	45	94	32	194	36
Total	2,266	100	289	100	543	100
**Place of residence**						
Rural	1,475	65	195	67	402	74
Urban	791	35	94	33	141	26
Total	2,266	100	289	100	543	100

### Econometric Results

Moving away from the descriptive analysis, we present the regression results in the following sections. The results are presented as marginal effects and shown in Table 4. It had been established through this analysis that being a beneficiary of any safety net program had a significant impact on the following coping mechanisms to shocks during Covid-19 pandemic; receiving assistance from friends and family, reducing food consumption and relying on savings. The first and final columns of the table indicate no significant impact of safety nets programs on household engagement in additional income generating activities as a coping mechanism and on failure to cope after experiencing a shock during the pandemic.

**Table 4:** Marginal effects-overall probit models.

	**Engaging in additional income generating activities**	**Receiving assistance from friends and family**	**Reducing food consumption**	**Relying on savings**	**Doing nothing**
Safety nets beneficiary (0/1)	0.00625 (0.013)	0.0486^**^ (0.019)	−0.0284^**^ (0.014)	−0.0517^***^ (0.016)	−0.0123 (0.037)
Age of head (log)	0.00704 (0.009)	0.00715 (0.008)	−0.0186 (0.019)	−0.00527 (0.018)	0.0399 (0.031)
Female (0/1)	−0.00560 (0.006)	0.0192^**^ (0.009)	−0.00750 (0.016)	−0.00842 (0.017)	0.0159 (0.029)
Household size (log)	0.0264^**^ (0.011)	−0.0180^*^ (0.011)	0.0864^***^ (0.026)	−0.000519 (0.026)	−0.0501 (0.043)
Dependents	−0.00390 (0.003)	0.00147 (0.003)	−0.0257^***^ (0.007)	0.000885 (0.006)	0.0119 (0.011)
Adults above 18 years (log)	−0.0232^***^ (0.009)	0.00442 (0.009)	−0.0603^***^ (0.021)	0.0117 (0.021)	0.0422 (0.036)
No education		−0.00312 (0.015)	0.0274 (0.025)	−0.0485 (0.037)	0.0945^*^ (0.049)
Secondary education	−0.00766 (0.006)	0.00240 (0.005)	0.00652 (0.012)	0.00113 (0.012)	−0.0350^*^ (0.020)
Tertiary education	0.00941 (0.010)	−0.0208 (0.013)	0.00103 (0.021)	0.0273 (0.019)	−0.0922^***^ (0.035)
Married (0/1)	0.00489 (0.006)	−0.000611 (0.006)	0.0101 (0.016)	0.00214 (0.017)	−0.0156 (0.028)
Agricultural sector (0/1)	−0.00101 (0.005)	−0.00680 (0.005)	−0.0262^**^ (0.010)	−0.0563^***^ (0.011)	0.0828^***^ (0.021)
Wealth quintile 2	0.0152^**^ (0.006)	−0.00114 (0.006)	−0.0229 (0.015)	0.00312 (0.015)	−0.0159 (0.024)
Wealth quintile 3	0.0178^**^ (0.007)	0.000517 (0.007)	−0.0490^***^ (0.014)	−0.0158 (0.016)	0.0137 (0.028)
Wealth quintile 4	0.0251^**^ (0.011)	0.00806 (0.009)	−0.0336^*^ (0.017)	−0.0175 (0.019)	0.0147 (0.033)
Wealth quintile 5	0.0117 (0.012)	−0.00164 (0.009)	0.00161 (0.027)	−0.0235 (0.021)	0.0161 (0.035)
Urban (0/1)	0.00901 (0.007)	0.00361 (0.005)	0.00287 (0.012)	−0.00648 (0.012)	−0.00139 (0.021)
Central region	−0.0417^***^ (0.012)	−0.0158^*^ (0.008)	−0.0588^***^ (0.019)	0.0474^***^ (0.014)	0.0495^*^ (0.027)
Southern region	−0.0430^***^ (0.012)	−0.0174^**^ (0.008)	0.00352 (0.021)	0.0372^***^ (0.013)	0.0367 (0.027)
Idiosyncratic shock (0/1)	−0.00618 (0.006)	0.00969 (0.006)	−0.0340^***^ (0.012)	0.0343^***^ (0.012)	0.0517^**^ (0.021)
Economic shock (0/1)	0.0269^***^ (0.004)	0.00313 (0.006)		0.0920^***^ (0.010)	0.363^***^ (0.019)
Health shock (0/1)	−0.00854 (0.006)	0.0128 (0.008)		−0.0600^***^ (0.012)	−0.0645^**^ (0.030)
Socio-political shock (0/1)	−0.0147^***^ (0.004)	−0.0126^***^ (0.004)		−0.0589^***^ (0.010)	0.0611^***^ (0.023)
*N*	3,048	3,140	1,638	3,140	3,140

*Standard errors in parentheses*.

* *p < 0.1*,

** *p < 0.05*,

*** *p < 0.01*.

As compared to a non-beneficiary household, a beneficiary household of any social safety net program was 5% more likely to rely on assistance from friends and family, 3% less likely to reduce food consumption, and 5% less likely to rely on savings in response to a shock.

Other factors that affected the household probability of adopting a specific coping mechanism included; sex of household head, household size, number of dependents in a household, number of household members above 18 years old, education of household head, sector of employment, and region. In addition to these variables, the scope as well as the type of shock experienced by a household also had an impact on the adoption of a specific coping mechanism. When hit by a shock, a female-headed household was about 2% more likely to receive assistance from friends and family as compared to a male-headed household, all things being the same.

As the household size increased, the household was more likely to engage in additional income-generating activities, less likely to receive assistance from friends and family, and more likely to reduce food consumption in response to a shock. On top of that, a unit increase in the number of dependents in a household was associated with less likelihood of reducing food consumption as a coping mechanism to shocks, all things being equal. On top of that, an increase in household members above 18 years was associated with less likelihood of engaging in additional income generating activities, and less likelihood of reducing food consumption in response to a shock. In addition, households for non-educated heads were more likely to fail to cope with any shock during the pandemic than those with primary education. Whereas, household heads with secondary and tertiary education were less likely to fail to cope with the shocks than those with primary education, all things being the same.

Compared to those employed in the non-agricultural sector, those in the agriculture sector were less likely to reduce food consumption or rely on savings. On the other hand, these households were more likely to fail to cope, unlike those in the non-agricultural sector. Compared to those in the lowest quintile of wealth (the poorest); the affluent households were more likely to engage in other income generating activities in response to a shock. The latter were also less likely to reduce food consumption as a coping mechanism.

In terms of regions, households in the central region were more likely to respond to a shock by relying on savings and more likely to fail to cope than those in the northern region. Households in the southern region were also more likely to rely on savings when a shock hits as compared to those in the northern region, all things being the same. On the other hand, those in the central and southern regions were less likely to engage in additional income generating activities and receive assistance from friends and family as compared to those in the northern region. In addition, those in the central region were also less likely to reduce food consumption in response to a shock unlike those in the northern region.

Idiosyncratic shocks were more likely to trigger reliance on savings and less likely to reduce food consumption as coping responses to shocks compared with covariate shocks. On top of that, if hit by an idiosyncratic shock during the pandemic, a household was more likely to fail to cope as compared to being hit by a covariate shock. In terms of shock types, economic shocks were more likely to trigger engagement in additional income generating activities as a coping response, whereas socio-political shocks were less likely to trigger this coping mechanism. On top of that, economic shocks were more likely to trigger reliance on savings and a high likelihood of failure to cope. On the other hand, when faced with a health shock, a household was less likely to rely on savings or fail to cope. Besides, households that faced a socio-political shock were more likely to fail to cope with the shock but less likely to receive assistance from friends and family or rely on savings.

To assess heterogeneity in the results across regions, regional regressions were run and the results are as presented in Tables 5–7.

**Table 5:** Northern region regression results on safety nets.

	**Engaging in additional income generating activities**	**Receiving assistance from friends and family**	**Reducing food consumption**	**Relying on savings**	**Doing nothing**
Safety nets beneficiary	0.00598 (0.013)	0.0482^**^ (0.019)	−0.0253 (0.016)	−0.0521^***^ (0.016)	−0.0134 (0.036)
Controls	Yes	Yes	Yes	Yes	Yes
*N*	3,048	3,140	1,638	3,140	3,140

*Standard errors in parentheses*.

* *p < 0.1*,

** *p < 0.05*,

*** *p < 0.01*.

*Full table is in Appendix*.

**Table 6:** Central region regression results on safety nets.

	**Engaging in additional income generating activities**	**Receiving assistance from friends and family**	**Reducing food consumption**	**Relying on savings**	**Doing nothing**
Safety nets beneficiary	0.00412 (0.012)	0.0489^**^ (0.019)	−0.0284^**^ (0.014)	−0.0515^***^ (0.016)	−0.0119 (0.037)
Controls	Yes	Yes	Yes	Yes	Yes
*N*	3,048	3,140	1,638	3,140	3,140

*Standard errors in parentheses*.

* *p < 0.1*,

** *p < 0.05*,

*** *p < 0.01*.

*Full table is in Appendix*.

**Table 7:** Southern region regression results on safety nets.

	**Engaging in additional income generating activities**	**Receiving assistance from friends and family**	**Reducing food consumption**	**Relying on savings**	**Doing nothing**
Safety nets beneficiary	0.00745 (0.013)	0.0508^**^ (0.020)	−0.0255 (0.016)	−0.0524^***^ (0.016)	−0.0139 (0.036)
Controls	Yes	Yes	Yes	Yes	Yes
*N*	3,048	3,140	1,638	3,140	3,140

*Standard errors in parentheses*.

* *p < 0.1*,

** *p < 0.05*,

*** *p < 0.01*.

*Full table is in Appendix*.

We found safety nets to be associated with a high likelihood of receiving assistance from friends and family as a household coping mechanism to shocks across all three regions. However, the results showed a significantly larger difference in the likelihood of seeking assistance between beneficiaries of social protection programs and non-beneficiaries in the southern region than the differences that existed in the other regions. There was also a significantly larger impact in the southern region. The beneficiary's likelihood of relying on savings was much lower than that of the non-beneficiary compared to the other regions.

A significantly large difference in the likelihood of reducing food consumption as a shock response also existed in the central region, unlike the other regions. In the area, any social protection program beneficiary was about 3% less likely to reduce food consumption in response to a shock than a non-beneficiary. Whereas, in the other regions, the probabilities' differences were not statistically significant. Full regional regression tables are in the Appendix.

Heterogeneity across genders was also assessed, and the results are as presented in Tables 8, 9

**Table 8:** Male head regression results on safety nets.

	**Engaging in additional income generating activities**	**Receiving assistance from friends and family**	**Reducing food consumption**	**Relying on savings**	**Doing nothing**
Safety nets beneficiary	0.00625 (0.013)	0.0486^**^ (0.019)	−0.0284^**^ (0.014)	−0.0517^***^ (0.016)	−0.0123 (0.037)
Controls	Yes	Yes	Yes	Yes	Yes
*N*	3,048	3,140	1,638	3,140	3,140

*Standard errors in parentheses*.

* *p < 0.1*,

** *p < 0.05*,

*** *p < 0.01*.

*Full table is in Appendix*.

**Table 9:** Female head regression results on safety nets.

	**Engaging in additional income generating activities**	**Receiving assistance from friends and family**	**Reducing food consumption**	**Relying on savings**	**Doing nothing**
Safety nets beneficiary	0.00625 (0.013)	0.0486^**^ (0.019)	−0.0284^**^ (0.014)	−0.0517^***^ (0.016)	−0.0123 (0.037)
Controls	Yes	Yes	Yes	Yes	Yes
*N*	3,048	3,140	1,638	3,140	3,140

*Standard errors in parentheses*.

* *p < 0.1*,

** *p < 0.05*,

*** *p < 0.01*.

*Full table is in Appendix*.

Gender regressions showed safety nets having a significant impact on receiving assistance from friends and family, reducing food consumption, and relying on savings for both households headed by males and females. The results showed no differences across the genders. Full gender regression tables are also in the Appendix.

## Discussion

Interesting results emanate from the study. Firstly, we established that households that benefited from various safety nets programs during the pandemic were less likely to reduce food consumption and rely on savings. Thus, the findings support what was also established in Ethiopia and other African countries (21). This may suggest a positive development, given that by affecting households in that way, it may help them accumulate some wealth, thereby reducing some perpetual poverty. Even though such a positive development was observed, the safety net recipients were also more likely to rely on remittances from friends and family. The results appear to be contrary to what we may expect in normalcy. Furthermore, the result is in contrast to what was established in other studies where it has been shown that the safety nets are associated with reduced dependence on partners and relatives compared to not having the safety nets (22).

Based on the above, we may speculate that this was such a case since most of the times, the households that are included in these programs hardly make enough incomes from which they can save (23, 24). When hit by any shock, such households rely more on assistance, whether formal safety net programs or informal remittances from friends and family. A finding that supports what was established in the context of other shocks in the Philippines and other developing countries, where these coping acted as insurance (25). Furthermore, the results also imply that the assistance being received was mainly being channeled into household consumption and not necessarily into household investment since we did not find evidence that the beneficiaries were more likely to engage in additional income generating activities than non-beneficiaries. This finding, therefore, is in line with the literature that established the cushioning effect of these safety nets (26–28). This may be because most of the targeted beneficiaries were urban poor, who were probably living hand to mouth situation, and that Covid-19 exacerbated the situation.

Apart from the result narrated, education also played an important role. We noted that households headed by an uneducated member were more likely to fail to cope with the shocks during the pandemic. Most of the time, they hardly have reliable incomes or connections to enable them to use other coping mechanisms. On the other hand, those with secondary and tertiary education had enough financial as well as human capital that enabled them to cope with the shocks in one way or the other. These findings were contrary to those by (29) who found that household head's education was not significantly related to any specific shock response behavior.

In this study, those in the third and fourth quintiles of wealth (the rich) were less likely to reduce food consumption in response to a shock than those in the lowest quintile (the poor). This supports the findings by (13) and can be explained by the fact that the former make enough incomes from other income generating activities enabling them to cover for their food consumption needs during a shock unlike the poor. This was also evidenced from the results that those in the second to the upper quintiles were more likely to engage in additional income generating activities in response to shocks as compared to the poorest since they have enabling financial and other resources.

The impact of household demographic characteristics implies that a household would reduce its food consumption in response to a shock if its size increased unless the additional member was a dependent. This is plausible as dependents have a lot of nutritional requirements, unlike others. And also, the household was likely to engage in additional income-generating activities if the additional member was below the age of 18. This is so since such members require a lot of support from the elder members, thereby forcing them to look for other activities where they can generate income from.

The finding that idiosyncratic shocks were more likely to trigger failure to cope was in contrast with a risk-sharing theory which stipulates that households have a wide range of coping mechanisms during an idiosyncratic shock unlike a covariate shock (30). On the other hand, this was plausible during this pandemic since many households had been affected by at least one shock, which made it hard for households to use other mechanisms of borrowing or seeking assistance from friends and family, rather they relied more on their own savings. This means that each household was in this pandemic and facing its impacts alone.

The paper has also established that there are regional differences in terms of coping mechanisms adopted by households during the pandemic. Unlike in other regions, there is a significant impact of safety nets on reduction of food consumption as a coping mechanism to shocks by households in the central region. Beneficiaries in this region are less likely to reduce food consumption as a shock response than non-beneficiaries. This may be explained by the fact that the highest proportion (55.8%) of people in the central region are poor unlike the other regions (31). Malawi Poverty Estimates 2020 by the NSO also established that the central region has the highest ultra-poverty rate (25.4%) and poverty is also deeper in this region at 20.1% as compared to other regions. Therefore, any levels of remittances in this region may likely have a significant impact on household food consumption unlike in other regions. We recognize the limitations of the study. First, the study is based on recall data, and hence bias may be an issue. Secondly, the methodology used does not address endogeneity and should be interpreted in the current methodology (32–34). Given our results, these results have some implications for research. Firstly, future studies should try to use other quasi-experimental methods such as an instrumental variable approach to see if the findings remain robust to the method of estimation. In our case, the lack of a proper instrument in the data made this a problem for us. Despite these limitations, the study has important implications for policy. The government of Malawi should increase the level and size of the transfers being used as they benefit the recipients, but they are not adequate to enable them to invest.

## Conclusion

We have established through this study a significant impact of safety nets on the increasing probability of household reliance on remittances from friends and family and decreasing the probability of food reduction and reliance on savings as coping mechanisms to shocks. The safety nets programs during Covid-19 pandemic likely improved the beneficiaries' nutrition status. However, these programs had no significant impact on household engagement in other income generating activities, as well as households' failure to cope. This implies they had no impact on household investment. Therefore, these safety net programs during the pandemic have to be scaled up. The amount of funds has to be revised upwards to enable households to have enough incomes to cover both consumption and investment needs. This will in turn, make vulnerable households self-reliant and reduce their dependency on remittances from friends and family during a crisis as this mechanism also has a negative impact on the incomes of the households rendering this assistance, especially during this pandemic as almost every household had been affected in one way or the other.

In addition to that, upon investing, vulnerable households may be able to accumulate enough asset holdings and build resilience to future shocks. They may eventually graduate from these social protection programs, and new vulnerable households will be able to be recruited and assisted likewise. In turn, the goal of poverty eradication will be achieved in Malawi.

## Data Availability Statement

The original contributions presented in the study are included in the article/Supplementary Materials. The data is available for free download at https://microdata.worldbank.org/index.php/catalog/3766.

## Author Contributions

All authors listed have made a substantial, direct, and intellectual contribution to the work and approved it for publication.

## Conflict of Interest

The authors declare that the research was conducted in the absence of any commercial or financial relationships that could be construed as a potential conflict of interest.

## Publisher's Note

All claims expressed in this article are solely those of the authors and do not necessarily represent those of their affiliated organizations, or those of the publisher, the editors and the reviewers. Any product that may be evaluated in this article, or claim that may be made by its manufacturer, is not guaranteed or endorsed by the publisher.

## References

[1] Chirwa GC Dulani B Sithole L Chunga JJ Alfonso W Tengatenga J. Malawi at the crossroads: does the fear of contracting covid-19 affect the propensity to vote? Euro J Dev Res. (2021). 10.1057/s41287-020-00353-1. [Epub ahead of print]. 33424140 PMC7781184

[2] Bonaccorsi G Pierri F Cinelli M Flori A Galeazzi A Porcelli F. Economic and social consequences of human mobility restrictions under COVID-19. Proc Natl Acad Sci USA. (2020) 117:15530–5. 10.1073/pnas.2007658117 32554604 PMC7355033

[3] Caggiano G Castelnuovo E Kima R. The global effects of Covid-19-induced uncertainty. Econ Lett. (2020) 194:109392. 10.1016/j.econlet.2020.109392 32834236 PMC7340019

[4] Fornaro L Wolf M. Covid-19 Coronavirus Macroeconomic Policy (SSRN Scholarly Paper ID 3560337). Social Science Research Network (2020). Available online at: https://papers.ssrn.com/abstract=3560337

[5] National Statistical Office World Bank. Findings-From-the-Second-Round-of-the-High-Frequency-Phone-Survey.pdf. (2020). Available online at: https://openknowledge.worldbank.org/bitstream/handle/10986/34545/Findings-from-the-Second-Round-of-the-High-Frequency-Phone-Survey.pdf?sequence=1&isAllowed=y (accessed May 10, 2021).

[6] National Statistical Office World Bank. Findings-From-the-Seventh-Round-of-the-High-Frequency-Phone-Survey.pdf. (2021). Available online at: https://openknowledge.worldbank.org/bitstream/handle/10986/35335/Findings-from-the-Seventh-Round-of-the-High-Frequency-Phone-Survey.pdf?sequence=1&isAllowed=y (accessed May 10, 2021).

[7] United Nations. Agenda for Sustainable Development. (2015). Available online at: https://sustainabledevelopment.un.org/content/documents/21252030%20Agenda%20for%20Sustainable%20Development%20web.pdf (accessed May 10, 2021).

[8] African Union Commission. Agenda 2063 the Africa We Want. (2015). Available online at: https://au.int/sites/default/files/documents/36204-doc-agenda2063_popular_version_en.pdf (accessed May 10, 2021).

[9] Government of Malawi. Malawi Growth and Development Strategy III. (2017). Available online at: https://npc.mw/wp-content/uploads/2020/07/MGDS_III.pdf (accessed May 10, 2021).

[10] Government of Malawi. Malawi Vision 2063. (2020). Available online at: https://malawi.un.org/sites/default/files/2021-01/MW2063-%20Malawi%20Vision%202063%20Document.pdf (accessed May 10, 2021).

[11] Bonfrer I Gustafsson-Wright E. Health shocks, coping strategies and foregone healthcare among agricultural households in Kenya. Global Public Health. (2017) 12:1369–90. 10.1080/17441692.2015.1130847 26785165

[12] Nguyen T-T Nguyen TT Grote U. Multiple shocks and households' choice of coping strategies in rural Cambodia. Ecol Econ. (2020) 167:106442. 10.1016/j.ecolecon.2019.106442

[13] Sparrow R Van de Poel E Hadiwidjaja G Yumna A Warda N Suryahadi A. Financial consequences of ill health and informal coping mechanisms in Indonesia. In: SMERU Research Institute Working Paper. Jakarta (2013).

[14] Yilma Z Mebratie A Sparrow R Abebaw D Dekker M Alemu G. Coping with shocks in rural Ethiopia. J Dev Stud. (2014) 50:1009–24. 10.1080/00220388.2014.909028

[15] Janssens W Pradhan MP De Groot R Sidze E Donfouet H Abajobir A. The short-term economic effects of COVID-19 and risk-coping strategies of low-income households in Kenya: a rapid analysis using weekly financial household data. In: Tinbergen Institute Disucssion Paper, 40. (2020). 10.2139/ssrn.3640340

[16] Adesina-Uthman GA Obaka AI. Financial coping strategies of households during COVID-19 induced lockdown. Empirical Econ Rev. (2020) 3:83–114. 10.29145/eer/32/030205

[17] Alderman H Yemtsov R. How can safety nets contribute to economic growth? World Bank Econ Rev. (2014) 28:1–20. 10.1093/wber/lht011

[18] Begum I Akter S Alam M Rahmatullah N. Social Safety Nets and Productive Outcomes: Evidence and Implications for Bangladesh. Bangladesh Agricultural University, Mymensingh, Bangladesh (2014).

[19] Hidrobo M Hoddinott J Peterman A Margolies A Moreira V. Cash, food, or vouchers? Evidence from a randomized experiment in northern Ecuador. J Dev Econ. (2014) 107:144–56. 10.1016/j.jdeveco.2013.11.009

[20] Hidrobo M Hoddinott J Kumar N Olivier M. Social protection, food security, asset formation. World Dev. (2018) 101:88–103. 10.1016/j.worlddev.2017.08.014

[21] Gilligan DO Hoddinott J Taffesse AS. The impact of Ethiopia's productive safety net programme and its linkages. J Dev Stud. (2009) 45:1684–706. 10.1080/00220380902935907

[22] Peterman A Neijhoft (Naomi) A Cook S Palermo TM. Understanding the linkages between social safety nets and childhood violence: a review of the evidence from low- and middle-income countries. Health Policy Plann. (2017) 32:1049–71. 10.1093/heapol/czx033 28444197 PMC5886196

[23] Ottie-Boakye D. Coverage of non-receipt of cash transfer (Livelihood Empowerment Against Poverty) and associated factors among older persons in the Mampong Municipality, Ghana – a quantitative analysis. BMC Geriatr. (2020) 20:406. 10.1186/s12877-020-01786-3 33059608 PMC7566032

[24] Jimu IM Msilimba G. Targeting practices and biases in social cash transfers: Experiences in rural Malawi. Afr Dev. (2018) 43:65–84.

[25] Adams RH Jr. Evaluating the economic impact of international remittances on developing countries using household surveys: a literature review. J Dev Stud. (2011) 47:809–28. 10.1080/00220388.2011.56329910123385

[26] Combes J-L Ebeke C. Remittances and household consumption instability in developing countries. World Dev. (2011) 39:1076–89. 10.1016/j.worlddev.2010.10.006

[27] De Janvry A Finan F Sadoulet E Vakis R. Can conditional cash transfer programs serve as safety nets in keeping children at school and from working when exposed to shocks? J Dev Econ. (2006) 79:349–73. 10.1016/j.jdeveco.2006.01.013

[28] Dercon S. Income risk, coping strategies, and safety nets. World Bank Res Obs. (2002) 17:141–66. 10.1093/wbro/17.2.1417994401

[29] Börner J Shively G Wunder S Wyman M. How do rural households cope with economic shocks? Insights from global data using hierarchical analysis. J Agric Econ. (2015) 66:392–414. 10.1111/1477-9552.12097

[30] Hess GD Shin K. Risk sharing by households within and across regions and industries. J Monet Econ. (2000) 45:533–60. 10.1016/S0304-3932(00)00007-6

[31] Government of Malawi. Malawi Poverty Report 2020. (2021). Available online at: https://microdata.worldbank.org/index.php/catalog/3818/download/51154

[32] Khandker SR Koolwal GB Samad HA. Handbook on impact evaluation: Quantitative methods and practices. World Bank Publ. (2009). 10.1596/978-0-8213-8028-4

[33] Palmer-Jones R. Handbook on impact evaluation: quantitative methods and practices, by SR Khandker, GB Koolwal and HA Samad. J Dev Effect. (2010) 2:387–90. 10.1080/19439342.2010.499188

[34] Angrist JD Pischke J-S. Mostly Harmless Econometrics. Princeton, NJ: Princeton University Press (2008).

