# Supplementary material for: Impact of Safety Nets on Household Coping Mechanisms for COVID-19 Pandemic in Malawi
Source: Front Public Health. 2022 Feb 7;9:806738. doi: 10.3389/fpubh.2021.806738 (PMC8858801; doi:10.3389/fpubh.2021.806738)
Supplement: Supplementary file 1 [file Data_Sheet_1.docx]

**APPENDICES**

Appendix 1: Pooled OLS full regression results

|  | Engaging in additional income generating activities | Receiving assistance from friends and family | Reducing food consumption | Relying on savings | Doing nothing |
| --- | --- | --- | --- | --- | --- |
|  |  |  |  |  |  |
| Safety Nets beneficiary (0/1) | 0.00393 | 0.0467^**^ | -0.0105 | -0.0393^***^ | -0.00874 |
|  | (0.011) | (0.019) | (0.010) | (0.015) | (0.037) |
| Age of head (log) | 0.00793 | 0.0110 | -0.00449 | -0.00813 | 0.0425 |
|  | (0.009) | (0.011) | (0.010) | (0.018) | (0.031) |
| Female (0/1) | -0.00569 | 0.0215^**^ | -0.00465 | -0.00984 | 0.0162 |
|  | (0.006) | (0.011) | (0.010) | (0.017) | (0.029) |
| Household size (log) | 0.0234^**^ | -0.0178 | 0.0385^***^ | 0.000876 | -0.0504 |
|  | (0.010) | (0.014) | (0.014) | (0.025) | (0.042) |
| Dependents | -0.00342 | 0.000785 | -0.0108^***^ | 0.000318 | 0.0114 |
|  | (0.003) | (0.003) | (0.004) | (0.006) | (0.011) |
| Adults above 18 years (log) | -0.0229^***^ | 0.00407 | -0.0286^**^ | 0.0126 | 0.0446 |
|  | (0.009) | (0.012) | (0.014) | (0.020) | (0.036) |
| No education | -0.0249^***^ | -0.00767 | 0.0103 | -0.0418^*^ | 0.0925^*^ |
|  | (0.006) | (0.012) | (0.018) | (0.024) | (0.052) |
| Secondary education | -0.00711 | 0.00208 | 0.00255 | 0.00323 | -0.0359^*^ |
|  | (0.006) | (0.005) | (0.007) | (0.012) | (0.021) |
| Tertiary education | 0.00699 | -0.0112^*^ | -0.00535 | 0.0404^*^ | -0.0832^**^ |
|  | (0.010) | (0.006) | (0.012) | (0.024) | (0.032) |
| Married (0/1) | 0.00365 | 0.000228 | 0.00457 | -0.00285 | -0.0167 |
|  | (0.005) | (0.010) | (0.009) | (0.018) | (0.028) |
| Agricultural sector (0/1) | -0.00124 | -0.00866^*^ | -0.0164^***^ | -0.0590^***^ | 0.0890^***^ |
|  | (0.006) | (0.005) | (0.006) | (0.012) | (0.022) |
| Wealth quintile2 | 0.0142^**^ | 0.00266 | -0.0136^*^ | 0.00800 | -0.0157 |
|  | (0.007) | (0.007) | (0.008) | (0.016) | (0.024) |
| Wealth quintile3 | 0.0179^**^ | 0.00281 | -0.0269^***^ | -0.0116 | 0.0164 |
|  | (0.008) | (0.008) | (0.008) | (0.017) | (0.029) |
| Wealth quintile4 | 0.0230^**^ | 0.0143 | -0.0162 | -0.0182 | 0.0168 |
|  | (0.010) | (0.012) | (0.010) | (0.019) | (0.033) |
| Wealth quintile5 | 0.0116 | 0.00242 | 0.00280 | -0.0154 | 0.0208 |
|  | (0.011) | (0.010) | (0.014) | (0.022) | (0.037) |
| Urban (0/1) | 0.00851 | 0.00705 | -0.000750 | -0.00636 | 0.000970 |
|  | (0.006) | (0.007) | (0.007) | (0.013) | (0.021) |
| Central region | -0.0324^***^ | -0.0218^**^ | -0.0288^***^ | 0.0429^***^ | 0.0485^*^ |
|  | (0.010) | (0.011) | (0.008) | (0.015) | (0.026) |
| Southern region | -0.0347^***^ | -0.0240^**^ | 0.00545 | 0.0312^**^ | 0.0385 |
|  | (0.010) | (0.011) | (0.009) | (0.014) | (0.026) |
| Idiosyncratic shock (0/1) | -0.00406 | 0.00997 | -0.0190^**^ | 0.0367^**^ | 0.0440^*^ |
|  | (0.007) | (0.006) | (0.008) | (0.014) | (0.023) |
| Economic shock (0/1) | 0.0276^***^ | 0.00200 | 0.0511^***^ | 0.0995^***^ | 0.356^***^ |
|  | (0.006) | (0.006) | (0.007) | (0.013) | (0.022) |
| Health shock (0/1) | -0.0113 | 0.0195^*^ | -0.0139^***^ | -0.0693^***^ | -0.0968^***^ |
|  | (0.007) | (0.012) | (0.004) | (0.013) | (0.035) |
| Socio-political shock (0/1) | -0.0160^***^ | -0.0136^***^ | -0.0268^***^ | -0.0765^***^ | 0.0520^**^ |
|  | (0.006) | (0.005) | (0.004) | (0.012) | (0.026) |
| _cons | -0.0177 | 0.000911 | 0.0327 | 0.0267 | -0.0726 |
|  | (0.038) | (0.038) | (0.040) | (0.072) | (0.127) |
| *N* | 3140 | 3140 | 3140 | 3140 | 3140 |
|  |  |  |  |  |  |

Standard errors in parentheses

^*^ *p* < 0.1, ^**^ *p* < 0.05, ^***^ *p* < 0.01

Appendix 2: Northern region full regression results

|  | Engaging in additional income generating activities | Receiving assistance from friends and family | Reducing food consumption | Relying on savings | Doing nothing |
| --- | --- | --- | --- | --- | --- |
|  |  |  |  |  |  |
| Safety Nets beneficiary (0/1) | 0.00598 | 0.0482^**^ | -0.0253 | -0.0521^***^ | -0.0134 |
|  | (0.013) | (0.019) | (0.016) | (0.016) | (0.036) |
| Age of head (log) | 0.00697 | 0.00720 | -0.0186 | -0.00538 | 0.0399 |
|  | (0.009) | (0.008) | (0.020) | (0.018) | (0.031) |
| Female head (0/1) | -0.00571 | 0.0189^**^ | -0.00282 | -0.00942 | 0.0145 |
|  | (0.006) | (0.009) | (0.017) | (0.017) | (0.028) |
| Household size (log) | 0.0264^**^ | -0.0180^*^ | 0.0873^***^ | 0.0000251 | -0.0495 |
|  | (0.011) | (0.011) | (0.026) | (0.026) | (0.043) |
| Dependents | -0.00393 | 0.00145 | -0.0241^***^ | 0.000515 | 0.0115 |
|  | (0.003) | (0.003) | (0.007) | (0.006) | (0.011) |
| Adults above 18 years (log) | -0.0231^***^ | 0.00447 | -0.0640^***^ | 0.0125 | 0.0431 |
|  | (0.009) | (0.009) | (0.022) | (0.021) | (0.036) |
| No education |  | -0.00308 | 0.0199 | -0.0488 | 0.0940^*^ |
|  |  | (0.015) | (0.026) | (0.037) | (0.049) |
| Secondary education | -0.00764 | 0.00241 | 0.00462 | 0.00142 | -0.0346^*^ |
|  | (0.006) | (0.005) | (0.012) | (0.012) | (0.020) |
| Tertiary education | 0.00956 | -0.0209 | -0.00751 | 0.0281 | -0.0911^***^ |
|  | (0.010) | (0.014) | (0.021) | (0.019) | (0.035) |
| Married (0/1) | 0.00485 | -0.000675 | 0.0114 | 0.00167 | -0.0157 |
|  | (0.006) | (0.006) | (0.015) | (0.017) | (0.028) |
| Agricultural sector (0/1) | -0.00103 | -0.00672 | -0.0276^***^ | -0.0562^***^ | 0.0828^***^ |
|  | (0.005) | (0.005) | (0.011) | (0.011) | (0.021) |
| Wealth quintile2 | 0.0153^**^ | -0.00100 | -0.0298^*^ | 0.00401 | -0.0147 |
|  | (0.006) | (0.006) | (0.016) | (0.015) | (0.024) |
| wealth quintile3 | 0.0179^**^ | 0.000560 | -0.0554^***^ | -0.0145 | 0.0151 |
|  | (0.008) | (0.007) | (0.015) | (0.016) | (0.028) |
| Wealth quintile4 | 0.0253^**^ | 0.00809 | -0.0409^**^ | -0.0163 | 0.0164 |
|  | (0.012) | (0.009) | (0.018) | (0.019) | (0.033) |
| Wealth quintile5 | 0.0119 | -0.00142 | -0.0104 | -0.0217 | 0.0191 |
|  | (0.012) | (0.009) | (0.027) | (0.021) | (0.035) |
| Urban (0/1) | 0.00922 | 0.00386 | -0.0101 | -0.00478 | 0.000364 |
|  | (0.006) | (0.005) | (0.012) | (0.012) | (0.021) |
| Northern region (0/1) | 0.0425^***^ | 0.0167^**^ | 0.0268 | -0.0422^***^ | -0.0430^*^ |
|  | (0.012) | (0.008) | (0.019) | (0.012) | (0.025) |
| Idiosyncratic shock (0/1) | -0.00632 | 0.00959 | -0.0305^***^ | 0.0341^***^ | 0.0511^**^ |
|  | (0.006) | (0.006) | (0.011) | (0.012) | (0.020) |
| Economic shock (0/1) | 0.0269^***^ | 0.00318 |  | 0.0919^***^ | 0.363^***^ |
|  | (0.004) | (0.006) |  | (0.010) | (0.019) |
| Health shock (0/1) | -0.00842 | 0.0130 |  | -0.0597^***^ | -0.0632^**^ |
|  | (0.006) | (0.008) |  | (0.012) | (0.030) |
| Socio-political shock (0/1) | -0.0146^***^ | -0.0125^***^ |  | -0.0582^***^ | 0.0620^***^ |
|  | (0.004) | (0.004) |  | (0.011) | (0.023) |
| *N* | 3048 | 3140 | 1638 | 3140 | 3140 |
|  |  |  |  |  |  |

Standard errors in parentheses

^*^ *p* < 0.1, ^**^ *p* < 0.05, ^***^ *p* < 0.01

Appendix 3: Central region full regression results

|  | Engaging in additional income generating activities | Receiving assistance from friends and family | Reducing food consumption | Relying on savings | Doing nothing |
| --- | --- | --- | --- | --- | --- |
|  |  |  |  |  |  |
| Safety Nets beneficiary | 0.00412 | 0.0489^**^ | -0.0284^**^ | -0.0515^***^ | -0.0119 |
|  | (0.012) | (0.019) | (0.014) | (0.016) | (0.037) |
| Age of head (log) | 0.00546 | 0.00718 | -0.0185 | -0.00400 | 0.0406 |
|  | (0.009) | (0.008) | (0.019) | (0.018) | (0.031) |
| Female head (0/1) | -0.00487 | 0.0196^**^ | -0.00762 | -0.00906 | 0.0154 |
|  | (0.006) | (0.009) | (0.016) | (0.017) | (0.029) |
| Household size (log) | 0.0258^**^ | -0.0193^*^ | 0.0863^***^ | -0.000642 | -0.0490 |
|  | (0.012) | (0.011) | (0.026) | (0.026) | (0.043) |
| Dependents | -0.00394 | 0.00178 | -0.0257^***^ | 0.000830 | 0.0115 |
|  | (0.003) | (0.003) | (0.007) | (0.006) | (0.011) |
| Adults above 18 years (log) | -0.0212^**^ | 0.00574 | -0.0604^***^ | 0.00965 | 0.0390 |
|  | (0.009) | (0.009) | (0.021) | (0.021) | (0.036) |
| No education |  | -0.00421 | 0.0273 | -0.0474 | 0.0964^**^ |
|  |  | (0.016) | (0.025) | (0.037) | (0.049) |
| Secondary education | -0.00702 | 0.00255 | 0.00653 | 0.000885 | -0.0356^*^ |
|  | (0.006) | (0.005) | (0.012) | (0.012) | (0.020) |
| Tertiary education | 0.00943 | -0.0222 | 0.00101 | 0.0279 | -0.0917^***^ |
|  | (0.010) | (0.014) | (0.021) | (0.019) | (0.035) |
| Married (0/1) | 0.00447 | -0.000539 | 0.0101 | 0.00298 | -0.0148 |
|  | (0.006) | (0.006) | (0.016) | (0.017) | (0.028) |
| Agricultural sector (0/1) | -0.00312 | -0.00774^*^ | -0.0261^**^ | -0.0553^***^ | 0.0841^***^ |
|  | (0.005) | (0.005) | (0.010) | (0.011) | (0.021) |
| Wealth quintile2 | 0.0167^***^ | 0.000439 | -0.0231 | 0.000796 | -0.0182 |
|  | (0.006) | (0.006) | (0.015) | (0.015) | (0.024) |
| Wealth quintile3 | 0.0194^**^ | 0.00161 | -0.0491^***^ | -0.0173 | 0.0128 |
|  | (0.008) | (0.007) | (0.014) | (0.016) | (0.028) |
| Wealth quintile4 | 0.0267^**^ | 0.00928 | -0.0337^*^ | -0.0185 | 0.0145 |
|  | (0.012) | (0.010) | (0.017) | (0.019) | (0.033) |
| Wealth quintile5 | 0.0104 | -0.00142 | 0.00168 | -0.0239 | 0.0161 |
|  | (0.011) | (0.009) | (0.027) | (0.021) | (0.036) |
| Urban (0/1) | 0.0113^*^ | 0.00489 | 0.00261 | -0.00918 | -0.00290 |
|  | (0.007) | (0.006) | (0.012) | (0.012) | (0.021) |
| Central region (0/1) | -0.00791^*^ | -0.00288 | -0.0616^***^ | 0.0195^*^ | 0.0219 |
|  | (0.005) | (0.005) | (0.010) | (0.011) | (0.018) |
| Idiosyncratic shock (0/1) | -0.00225 | 0.0114^*^ | -0.0344^***^ | 0.0308^**^ | 0.0490^**^ |
|  | (0.006) | (0.006) | (0.011) | (0.012) | (0.020) |
| Economic shock (0/1) | 0.0244^***^ | 0.00161 |  | 0.0939^***^ | 0.366^***^ |
|  | (0.004) | (0.006) |  | (0.010) | (0.018) |
| Health shock (0/1) | -0.00836 | 0.0129 |  | -0.0602^***^ | -0.0653^**^ |
|  | (0.006) | (0.009) |  | (0.012) | (0.030) |
| Socio-political shock (0/1) | -0.0139^***^ | -0.0123^***^ |  | -0.0598^***^ | 0.0598^***^ |
|  | (0.004) | (0.004) |  | (0.010) | (0.023) |
| *N* | 3048 | 3140 | 1638 | 3140 | 3140 |
|  |  |  |  |  |  |

Standard errors in parentheses

^*^ *p* < 0.1, ^**^ *p* < 0.05, ^***^ *p* < 0.01

Appendix 4: Southern region full regression results

|  | Engaging in additional income generating activities | Receiving assistance from friends and family | Reducing food consumption | Relying on savings | Doing nothing |
| --- | --- | --- | --- | --- | --- |
|  |  |  |  |  |  |
| Safety Nets beneficiary (0/1) | 0.00745 | 0.0508^**^ | -0.0255 | -0.0524^***^ | -0.0139 |
|  | (0.013) | (0.020) | (0.016) | (0.016) | (0.036) |
| Age of head (log) | 0.00637 | 0.00698 | -0.0187 | -0.00396 | 0.0408 |
|  | (0.009) | (0.008) | (0.019) | (0.018) | (0.031) |
| Female head (0/1) | -0.00331 | 0.0210^**^ | -0.00312 | -0.0116 | 0.0125 |
|  | (0.006) | (0.010) | (0.017) | (0.017) | (0.028) |
| Household size (log) | 0.0250^**^ | -0.0193^*^ | 0.0894^***^ | 0.000659 | -0.0474 |
|  | (0.011) | (0.011) | (0.027) | (0.026) | (0.043) |
| Dependents | -0.00347 | 0.00185 | -0.0256^***^ | -0.0000743 | 0.0106 |
|  | (0.003) | (0.003) | (0.007) | (0.006) | (0.011) |
| Adults above 18 years (log) | -0.0229^**^ | 0.00529 | -0.0607^***^ | 0.0111 | 0.0397 |
|  | (0.009) | (0.010) | (0.023) | (0.021) | (0.036) |
| No education |  | -0.00424 | 0.0256 | -0.0479 | 0.0960^**^ |
|  |  | (0.015) | (0.026) | (0.037) | (0.049) |
| Secondary education | -0.00735 | 0.00244 | 0.00514 | 0.00150 | -0.0351^*^ |
|  | (0.006) | (0.005) | (0.012) | (0.012) | (0.020) |
| Tertiary education | 0.00770 | -0.0216 | -0.00191 | 0.0300 | -0.0894^**^ |
|  | (0.010) | (0.013) | (0.021) | (0.019) | (0.035) |
| Married (0/1) | 0.00497 | -0.000278 | 0.0115 | 0.00202 | -0.0147 |
|  | (0.006) | (0.006) | (0.015) | (0.017) | (0.028) |
| Agricultural sector (0/1) | -0.00272 | -0.00794^*^ | -0.0280^***^ | -0.0549^***^ | 0.0846^***^ |
|  | (0.005) | (0.005) | (0.010) | (0.011) | (0.021) |
| Wealth quintile2 | 0.0156^**^ | -0.000467 | -0.0221 | 0.00239 | -0.0166 |
|  | (0.006) | (0.006) | (0.015) | (0.015) | (0.024) |
| Wealth quintile3 | 0.0175^**^ | 0.00118 | -0.0491^***^ | -0.0145 | 0.0151 |
|  | (0.008) | (0.007) | (0.014) | (0.016) | (0.028) |
| Wealth quintile4 | 0.0233^**^ | 0.00883 | -0.0338^*^ | -0.0158 | 0.0178 |
|  | (0.011) | (0.010) | (0.018) | (0.019) | (0.033) |
| Wealth quintile5 | 0.00803 | -0.00254 | -0.00458 | -0.0194 | 0.0219 |
|  | (0.011) | (0.009) | (0.026) | (0.021) | (0.036) |
| Urban (0/1) | 0.00842 | 0.00345 | 0.00295 | -0.00570 | 0.0000272 |
|  | (0.006) | (0.006) | (0.012) | (0.012) | (0.021) |
| Southern region (0/1) | -0.0108^**^ | -0.00626 | 0.0506^***^ | 0.00225 | -0.0000392 |
|  | (0.005) | (0.005) | (0.011) | (0.010) | (0.018) |
| Idiosyncratic shock (0/1) | -0.000907 | 0.0116^*^ | -0.0266^**^ | 0.0293^**^ | 0.0468^**^ |
|  | (0.006) | (0.006) | (0.011) | (0.012) | (0.020) |
| Economic shock (0/1) | 0.0244^***^ | 0.00159 |  | 0.0942^***^ | 0.367^***^ |
|  | (0.004) | (0.006) |  | (0.010) | (0.018) |
| Health shock (0/1) | -0.00982^*^ | 0.0117 |  | -0.0595^***^ | -0.0631^**^ |
|  | (0.006) | (0.008) |  | (0.012) | (0.030) |
| Socio-political shock (0/1) | -0.0149^***^ | -0.0128^***^ |  | -0.0585^***^ | 0.0612^***^ |
|  | (0.004) | (0.004) |  | (0.011) | (0.023) |
| *N* | 3048 | 3140 | 1638 | 3140 | 3140 |
|  |  |  |  |  |  |

Standard errors in parentheses

^*^ *p* < 0.1, ^**^ *p* < 0.05, ^***^ *p* < 0.01

Appendix 5 : Male head full regression results

|  | Engaging in additional income generating activities | Receiving assistance from friends and family | Reducing food consumption | Relying on savings | Doing nothing |
| --- | --- | --- | --- | --- | --- |
|  |  |  |  |  |  |
| Safety Nets beneficiary (0/1) | 0.00625 | 0.0486^**^ | -0.0284^**^ | -0.0517^***^ | -0.0123 |
|  | (0.013) | (0.019) | (0.014) | (0.016) | (0.037) |
| Age of head (log | 0.00704 | 0.00715 | -0.0186 | -0.00527 | 0.0399 |
|  | (0.009) | (0.008) | (0.019) | (0.018) | (0.031) |
| Male head (0/1) | 0.00560 | -0.0192^**^ | 0.00750 | 0.00842 | -0.0159 |
|  | (0.006) | (0.009) | (0.016) | (0.017) | (0.029) |
| Household size (log) | 0.0264^**^ | -0.0180^*^ | 0.0864^***^ | -0.000519 | -0.0501 |
|  | (0.011) | (0.011) | (0.026) | (0.026) | (0.043) |
| Dependents | -0.00390 | 0.00147 | -0.0257^***^ | 0.000885 | 0.0119 |
|  | (0.003) | (0.003) | (0.007) | (0.006) | (0.011) |
| Adults above 18 years (log) | -0.0232^***^ | 0.00442 | -0.0603^***^ | 0.0117 | 0.0422 |
|  | (0.009) | (0.009) | (0.021) | (0.021) | (0.036) |
| No education |  | -0.00312 | 0.0274 | -0.0485 | 0.0945^*^ |
|  |  | (0.015) | (0.025) | (0.037) | (0.049) |
| Secondary education | -0.00766 | 0.00240 | 0.00652 | 0.00113 | -0.0350^*^ |
|  | (0.006) | (0.005) | (0.012) | (0.012) | (0.020) |
| Tertiary education | 0.00941 | -0.0208 | 0.00103 | 0.0273 | -0.0922^***^ |
|  | (0.010) | (0.013) | (0.021) | (0.019) | (0.035) |
| Married (0/1) | 0.00489 | -0.000611 | 0.0101 | 0.00214 | -0.0156 |
|  | (0.006) | (0.006) | (0.016) | (0.017) | (0.028) |
| Agricultural sector (0/1) | -0.00101 | -0.00680 | -0.0262^**^ | -0.0563^***^ | 0.0828^***^ |
|  | (0.005) | (0.005) | (0.010) | (0.011) | (0.021) |
| Wealth quintile2 | 0.0152^**^ | -0.00114 | -0.0229 | 0.00312 | -0.0159 |
|  | (0.006) | (0.006) | (0.015) | (0.015) | (0.024) |
| Wealth quintile3 | 0.0178^**^ | 0.000517 | -0.0490^***^ | -0.0158 | 0.0137 |
|  | (0.007) | (0.007) | (0.014) | (0.016) | (0.028) |
| Wealth quintile4 | 0.0251^**^ | 0.00806 | -0.0336^*^ | -0.0175 | 0.0147 |
|  | (0.011) | (0.009) | (0.017) | (0.019) | (0.033) |
| Wealth quintile5 | 0.0117 | -0.00164 | 0.00161 | -0.0235 | 0.0161 |
|  | (0.012) | (0.009) | (0.027) | (0.021) | (0.035) |
| Urban (0/1) | 0.00901 | 0.00361 | 0.00287 | -0.00648 | -0.00139 |
|  | (0.007) | (0.005) | (0.012) | (0.012) | (0.021) |
| Central region | -0.0417^***^ | -0.0158^*^ | -0.0588^***^ | 0.0474^***^ | 0.0495^*^ |
|  | (0.012) | (0.008) | (0.019) | (0.014) | (0.027) |
| Southern region | -0.0430^***^ | -0.0174^**^ | 0.00352 | 0.0372^***^ | 0.0367 |
|  | (0.012) | (0.008) | (0.021) | (0.013) | (0.027) |
| Idiosyncratic shock (0/1) | -0.00618 | 0.00969 | -0.0340^***^ | 0.0343^***^ | 0.0517^**^ |
|  | (0.006) | (0.006) | (0.012) | (0.012) | (0.021) |
| Economic shock (0/1) | 0.0269^***^ | 0.00313 |  | 0.0920^***^ | 0.363^***^ |
|  | (0.004) | (0.006) |  | (0.010) | (0.019) |
| Health shock (0/1) | -0.00854 | 0.0128 |  | -0.0600^***^ | -0.0645^**^ |
|  | (0.006) | (0.008) |  | (0.012) | (0.030) |
| Socio-political shock (0/1) | -0.0147^***^ | -0.0126^***^ |  | -0.0589^***^ | 0.0611^***^ |
|  | (0.004) | (0.004) |  | (0.010) | (0.023) |
| *N* | 3048 | 3140 | 1638 | 3140 | 3140 |
|  |  |  |  |  |  |

Standard errors in parentheses

^*^ *p* < 0.1, ^**^ *p* < 0.05, ^***^ *p* < 0.01

Appendix 6: Female head full regression results

|  | Engaging in additional income generating activities | Receiving assistance from friends and family | Reducing food consumption | Relying on savings | Doing nothing |
| --- | --- | --- | --- | --- | --- |
|  |  |  |  |  |  |
| Safety Nets beneficiary (0/1) | 0.00625 | 0.0486^**^ | -0.0284^**^ | -0.0517^***^ | -0.0123 |
|  | (0.013) | (0.019) | (0.014) | (0.016) | (0.037) |
| Age of head (log) | 0.00704 | 0.00715 | -0.0186 | -0.00527 | 0.0399 |
|  | (0.009) | (0.008) | (0.019) | (0.018) | (0.031) |
| Female head (0/1) | -0.00560 | 0.0192^**^ | -0.00750 | -0.00842 | 0.0159 |
|  | (0.006) | (0.009) | (0.016) | (0.017) | (0.029) |
| Household size (log) | 0.0264^**^ | -0.0180^*^ | 0.0864^***^ | -0.000519 | -0.0501 |
|  | (0.011) | (0.011) | (0.026) | (0.026) | (0.043) |
| Dependents | -0.00390 | 0.00147 | -0.0257^***^ | 0.000885 | 0.0119 |
|  | (0.003) | (0.003) | (0.007) | (0.006) | (0.011) |
| Adults above 18 years (log) | -0.0232^***^ | 0.00442 | -0.0603^***^ | 0.0117 | 0.0422 |
|  | (0.009) | (0.009) | (0.021) | (0.021) | (0.036) |
| No education |  | -0.00312 | 0.0274 | -0.0485 | 0.0945^*^ |
|  |  | (0.015) | (0.025) | (0.037) | (0.049) |
| Secondary education | -0.00766 | 0.00240 | 0.00652 | 0.00113 | -0.0350^*^ |
|  | (0.006) | (0.005) | (0.012) | (0.012) | (0.020) |
| Tertiary education | 0.00941 | -0.0208 | 0.00103 | 0.0273 | -0.0922^***^ |
|  | (0.010) | (0.013) | (0.021) | (0.019) | (0.035) |
| Married (0/1) | 0.00489 | -0.000611 | 0.0101 | 0.00214 | -0.0156 |
|  | (0.006) | (0.006) | (0.016) | (0.017) | (0.028) |
| Agricultural sector (0/1) | -0.00101 | -0.00680 | -0.0262^**^ | -0.0563^***^ | 0.0828^***^ |
|  | (0.005) | (0.005) | (0.010) | (0.011) | (0.021) |
| Wealth quintile2 | 0.0152^**^ | -0.00114 | -0.0229 | 0.00312 | -0.0159 |
|  | (0.006) | (0.006) | (0.015) | (0.015) | (0.024) |
| Wealth quintile3 | 0.0178^**^ | 0.000517 | -0.0490^***^ | -0.0158 | 0.0137 |
|  | (0.007) | (0.007) | (0.014) | (0.016) | (0.028) |
| Wealth quintile4 | 0.0251^**^ | 0.00806 | -0.0336^*^ | -0.0175 | 0.0147 |
|  | (0.011) | (0.009) | (0.017) | (0.019) | (0.033) |
| Wealth quintile5 | 0.0117 | -0.00164 | 0.00161 | -0.0235 | 0.0161 |
|  | (0.012) | (0.009) | (0.027) | (0.021) | (0.035) |
| Urban (0/1) | 0.00901 | 0.00361 | 0.00287 | -0.00648 | -0.00139 |
|  | (0.007) | (0.005) | (0.012) | (0.012) | (0.021) |
| Central region | -0.0417^***^ | -0.0158^*^ | -0.0588^***^ | 0.0474^***^ | 0.0495^*^ |
|  | (0.012) | (0.008) | (0.019) | (0.014) | (0.027) |
| Southern region | -0.0430^***^ | -0.0174^**^ | 0.00352 | 0.0372^***^ | 0.0367 |
|  | (0.012) | (0.008) | (0.021) | (0.013) | (0.027) |
| Idiosyncratic shock (0/1) | -0.00618 | 0.00969 | -0.0340^***^ | 0.0343^***^ | 0.0517^**^ |
|  | (0.006) | (0.006) | (0.012) | (0.012) | (0.021) |
| Economic shock (0/1) | 0.0269^***^ | 0.00313 |  | 0.0920^***^ | 0.363^***^ |
|  | (0.004) | (0.006) |  | (0.010) | (0.019) |
| Health shock (0/1) | -0.00854 | 0.0128 |  | -0.0600^***^ | -0.0645^**^ |
|  | (0.006) | (0.008) |  | (0.012) | (0.030) |
| Socio-political shock (0/1) | -0.0147^***^ | -0.0126^***^ |  | -0.0589^***^ | 0.0611^***^ |
|  | (0.004) | (0.004) |  | (0.010) | (0.023) |
| *N* | 3048 | 3140 | 1638 | 3140 | 3140 |
|  |  |  |  |  |  |

Standard errors in parentheses

^*^ *p* < 0.1, ^**^ *p* < 0.05, ^***^ *p* < 0.01
